# European Psychiatry: 2025 in review

**DOI:** 10.1192/j.eurpsy.2026.10150

**Published:** 2026-01-23

**Authors:** Sophia Frangou, Umberto Volpe

**Affiliations:** 1 Department of Psychiatry, Icahn School of Medicine at Mount Sinai, New York, USA; 2 Universita Politecnica delle Marche, Italy

In 2025, *European Psychiatry* strengthened its position as a leading journal in the field. The last 12 months marked a period of continued growth, intellectual breadth, and sustained engagement with the evolving priorities of psychiatric research and practice. As the main voice of the European Psychiatric Association (EPA), the journal remains committed to the values of the Association, which are reflected in the journal’s core mission: to publish rigorous, clinically relevant, and methodologically sound research that advances understanding of mental health and aims to improve clinical care and patient outcomes.

The journal attracted 749 submissions in 2025 and accepted 165 manuscripts, highlighting both the strength of the submission pipeline and the breadth of high-quality work shared with the field. Indicators of the journal’s influence remained strong: the Journal Impact Factor (2024) was 6.7, ranking 18th out of 288 journals in its category, while full-text downloads exceeded 3.4 million, demonstrating broad engagement from readers worldwide. European Psychiatry acknowledges the growing influence of artificial intelligence (AI) on scientific research and scholarly publishing. In our recent editorial [1] we discuss how AI-enhanced tools are increasingly used at different stages of the publication process, including language editing and aspects of peer review and content analysis. The editorial outlines the journal’s perspective on these developments, weighing their potential benefits against the concerns raised within the research community, and sets out clear principles to support responsible practice in scientific writing and publishing.

We are grateful to the authors who entrusted us with their work. Research articles, EPA guidance, and position papers remain the backbone of the Journal’s content. Articles published in 2025 showcased the diversity of contemporary psychiatric research. Original research articles spanned clinical psychiatry, epidemiology, neuroscience, genetics, and psychological science. Many studies emphasized dimensional and transdiagnostic approaches, developmental trajectories, and the integration of biological, psychological, and social perspectives. These trends reflect broader shifts in the field toward comprehensive models of mental health and illness that transcend traditional diagnostic boundaries. Large datasets and advanced analytical methods featured prominently. Studies drawing on population cohorts, electronic health records, and multimodal imaging increased, alongside work using computational or complex statistical approaches. Clinical relevance remained a core focus. Many publications examined treatment outcomes, service delivery, and real-world patient populations. Research exploring disparities in mental health outcomes across demographic groups and studies addressing social and environmental determinants of mental health reinforced the journal’s role at the intersection of psychiatry, public health, and policy. Table 1 summarizes the papers with the highest number of downloads from Cambridge Core, the highest Altmetric scores, and the highest citation counts during 2025.

**Table 1.** Most influential papers in 2025

Most downloaded papers	No of downloads
The time has come for revising the rules of clozapine blood monitoring in Europe. A joint expert statement from the European Clozapine Task Force [2]	6203
Diagnosing ADHD in adults in randomized controlled studies: a scoping review [3]	4635
Neuropsychiatric adverse events associated with Glucagon-like peptide–1 receptor agonists: a pharmacovigilance analysis of the FDA Adverse Event Reporting System database [4]	3979
Use of psychedelic treatments in psychiatric clinical practice: an EPA policy paper [5]	3381
A whole-brain voxel-based analysis of structural abnormalities in PTSD: An ENIGMA-PGC study [6]	3149

Most attention in media and social media	Altmetric score
Sex differences in adults with attention-deficit/hyperactivity disorder: A population-based study [7]	148
Physical activity in older adults as a predictor of alcohol consumption – a longitudinal analysis of 3133 individuals in the SHARE study [8]	101
Effects of substituting TV-watching time with physical activities or sleep on incident major depression. Results from the lifelines cohort study [9]	98
Diagnosing ADHD in adults in randomized controlled studies: a scoping review [3]	87
The time has come for revising the rules of clozapine blood monitoring in Europe. A joint expert statement from the European Clozapine Task Force [2]	46

Most cited	No. of citations
Suicidal risk among adolescent psychiatric inpatients: the role of insomnia, depression, and social-personal factors [10]	37
The time has come for revising the rules of clozapine blood monitoring in Europe. A joint expert statement from the European Clozapine Task Force [2]	19
Neuropsychiatric adverse events associated with Glucagon-like peptide–1 receptor agonists: a pharmacovigilance analysis of the FDA Adverse Event Reporting System database [4]	16
Association between adverse childhood experiences and suicidal behavior in affective disorders: A systematic review and meta-analysis [11]	13
The effect of scopolamine on memory and attention: a systematic review and meta-analysis [12]	8

*Note:* Downloads from Cambridge Core (cambridge.org/core/journals/european-psychiatry/). Altmetric score from Altmetric Explorer (altmetric.com). Citations from Dimensions (*app.dimensions.ai*). Compiled 08 January 2026.

Peer review was central to the journal’s quality in 2025, and we are deeply grateful to the reviewers whose time, care, and expertise significantly strengthened the clarity and rigor of the manuscripts considered for publication. The journal also benefited from a diverse international authorship. Studies drew data from a wide range of health systems and settings, highlighting both shared challenges and region-specific issues in mental health.

At the end of 2025, Professor Andrea Fiorillo stepped down from his role as Editor as he began his term as President of the EPA. We warmly thank Professor Fiorillo for his outstanding service reflecting astute editorial judgment and commitment to the journal and its academic mission. In his place, we welcomed Professor Umberto Volpe as the new Co-Editor-in-Chief. Professor Volpe is Professor of Psychiatry at Università Politecnica delle Marche (Ancona), where he chairs the Unit of Clinical Psychiatry and directs the School of Specialization in Psychiatry. His research interests span digital and tele-mental health, care pathways, and psychiatric rehabilitation. He is an active member of the EPA and has served the Association in many roles, including his recent election to the EPA board.

Looking ahead, we aim to remain attentive to both progress and unmet needs across Europe while we continue broadening the journal’s content to reflect the diverse clinical and research priorities of the field. Two current calls for papers outline key priorities for the coming period (https://www.cambridge.org/core/journals/european-psychiatry/announcements/call-for-papers). First, we have a special call on “T*he transformative role of AI in psychiatry and mental health,”* welcoming submissions across topics such as AI-based diagnostic and predictive tools, treatment personalization, ethics and governance, and AI-enabled neuroimaging and computational psychiatry. Second, we also encourage submissions to the “Population Neuroscience Perspectives of Psychopathology” special collection, which aims to showcase how large-scale studies, particularly but not exclusively on neuroimaging and genetics, can advance understanding of the biological underpinnings of mental disorders. Submissions to both special collections may include original research, reviews, or viewpoints.

We enter 2026 with a clear commitment to rigorous, clinically relevant scholarship and to providing a forum for thoughtful debate as the field continues to evolve.
